# A novel case of glial transdifferentiation in renal medullary carcinoma brain metastasis

**DOI:** 10.1186/s40478-025-01929-w

**Published:** 2025-01-20

**Authors:** Maria A. Gubbiotti, Ian E. McCutcheon, Priya Rao, Giannicola Genovese, Linghua Wang, Artem Tarasov, Vladislav Putintsev, Amber Berlinski, Danil Stupichev, Kirill Kriukov, Suren Davitavyan, Basim Salem, Alexander Sarachakov, Dmitry Lebedev, Michael Hensley, Alexander Bagaev, Francesca Paradiso, Vladimir Kushnarev, Gleb Khegai, Nizar M. Tannir, Pavlos Msaouel

**Affiliations:** 1 Department of Pathology, The University of Texas MD Anderson Cancer Center, 1515 Holcombe Boulevard, Unit 85, Houston, TX 77030-3721 USA; 2 Department of Neurosurgery, University of Texas MD Anderson Cancer Center, Houston, TX USA; 3 Department of Genitourinary Medical Oncology, The University of Texas MD Anderson Cancer Center Unit 1374, 1155 Pressler St, Houston, TX 77030-3721 USA; 4 Department of Genomic Medicine, The University of Texas MD Anderson Cancer Center, Houston, TX USA; 5 David H. Koch Center for Applied Research of Genitourinary Cancers, The University of Texas, Anderson Cancer Center, Houston, MD, TX 77030 USA; 6 The University of Texas MD Anderson Cancer Center UTHealth Houston Graduate School of Biomedical Sciences (GSBS), Houston, TX 77030 USA; 7 BostonGene Corporation, Waltham, MA 02453 USA; 8 Department of Translational Molecular Pathology, The University of Texas MD Anderson Cancer Center, Houston, TX USA; 9 Department of Genitourinary Medical Oncology, Division of Cancer Medicine, The University of Texas MD Anderson Cancer Center Unit 1374, 1155 Pressler St, Houston, TX 77030-3721 USA

**Keywords:** Renal medullary carcinoma, INI1, SMARCB1, Glial transdifferentiation, Tazemetostat, EZH2, H3K27me3

## Abstract

Renal medullary carcinoma is a rare undifferentiated tumor of the kidney associated with sickle cell trait and characterized by INI1 (SMARCB1) loss. Although metastasis to lungs, lymph nodes, and bone is commonly reported, distant spread to the central nervous system almost never occurs. Here we present an unusual case of a patient with renal medullary carcinoma with metastasis to the brain following treatment which included tazemetostat, an EZH2 inhibitor. The metastatic brain lesion harbored morphologic, immunohistochemical, and methylation profile supportive of a primary CNS phenotype with loss of the trimethylated lysine 27 residue of histone 3 while maintaining INI1 loss and a specific gene fusion shared with the patient’s tumor prior to initiation of tazemetostat therapy. Therefore, given the common genetic signatures in the brain metastasis and the patient’s prior tumor, this case represents a rare event of glial transdifferentiation in a brain metastasis of renal medullary carcinoma following the use of an epigenetic modulator. As renal medullary carcinoma has been known to cleverly utilize adaptive mechanisms for survival, we propose that such cell plasticity seen in this case may have been provoked by the use of a drug that alters the epigenetic signature of the tumor cells. Thus, careful assessment of tumor biology following novel therapeutic treatment options must be performed in order to note such unexpected consequences of treatment.

**Supplementary Information:**

The online version contains supplementary material available at 10.1186/s40478-025-01929-w.

## Introduction

Renal medullary carcinoma (RMC) is a rare neoplasm of the kidney associated with sickle cell trait [25, 34]. The defining molecular feature is loss of INI1 (also known as SMARCB1) as identified by immunohistochemistry [28]. High-intensity exercise in individuals with sickle cell trait may aggravate renal medullary hypoxia, which can result in INI1 degradation and increased RMC risk [33, 34]. RMC is highly aggressive and almost always presents with metastatic disease most commonly in the lymph nodes and lungs [23], resulting in a median survival of only four months from diagnosis when the patient is left untreated [6]. However, outcomes are steadily improving with the establishment of effective first-line [26], and more recently second- and third-line regimens [39, 40] that have improved median survival to at least 18 months from diagnosis [23]. Tazemetostat is an oral small-molecule inhibitor of the Enhancer of Zeste Homolog 2 (EZH2) methyltransferase responsible for tri-methylating lysine 27 on histone H3 (H3K27me3), which plays a critical role in modifying the epigenetic landscape of cells [18]. More specifically the H3K27me3 mark added by EZH2 suppresses gene expression. EZH2 inhibition by tazemetostat reduces the levels of H3K27me3, leading to a loosening of chromatin structure in specific regions of the genome that EZH2 typically represses [20]. INI1 loss leads to EZH2 upregulation that promotes tumor growth [18, 22]. Tazemetostat has accordingly demonstrated efficacy and received FDA approval for the treatment of INI1-deficient epithelioid sarcoma [10]. However, experience with tazemetostat in RMC remains scarce with only one case reported in the literature to date [4].

Notably, despite its aggressive tendency for distant metastasis, RMC rarely spreads to the central nervous system (CNS) with only 3.1% of patients presenting with brain metastases at diagnosis and initiation of systemic therapy [23]. In contrast, approximately 8% of patients with other renal cell carcinoma subtypes have brain metastases at the time of systemic therapy initiation [35]. Certain rare CNS neoplasms share INI1 loss with RMC. Examples include atypical teratoid/rhabdoid tumor (AT/RT), cribriform neuroepithelial tumor (CRINET), low-grade diffusely infiltrative tumor (LGDIT) INI1-mutant [13], and desmoplastic myxoid tumor of the pineal region. Though the INI1-deficient malignant rhabdoid tumor can co-occur with AT/RT in the context of rhabdoid tumor predisposition syndrome in patients with *SMARCB1* germ line variant *RPTS1*, RMC has never been reported to co-occur with AT/RT.

The concept of tumor cell transdifferentiation has been previously explored [8, 29]. In the context of tumor plasticity, the phenomenon of transdifferentiation, for example from luminal to basal subtype in breast cancer or proneural to mesenchymal phenotype in glioblastoma, acts as an adaptive response for tumor survival in unique microenvironments [1, 7, 11, 16]. Even rarer is the transdifferentiation of one tumor type to another tumor type such as angiosarcomatous transdifferentiation identified in melanoma or myeloid mimicry reported in gliomas [9, 17].Though sparse reports of such mimicry are present in the literature, to date, no cases of transdifferentiation following treatment with an epigenetic modulator can be found. Here we report the first case of RMC with glial mimicry/transdifferentiation of brain metastasis in a patient treated with the EZH2 inhibitor, tazemetostat.

## Case presentation

**Fig. 1:**
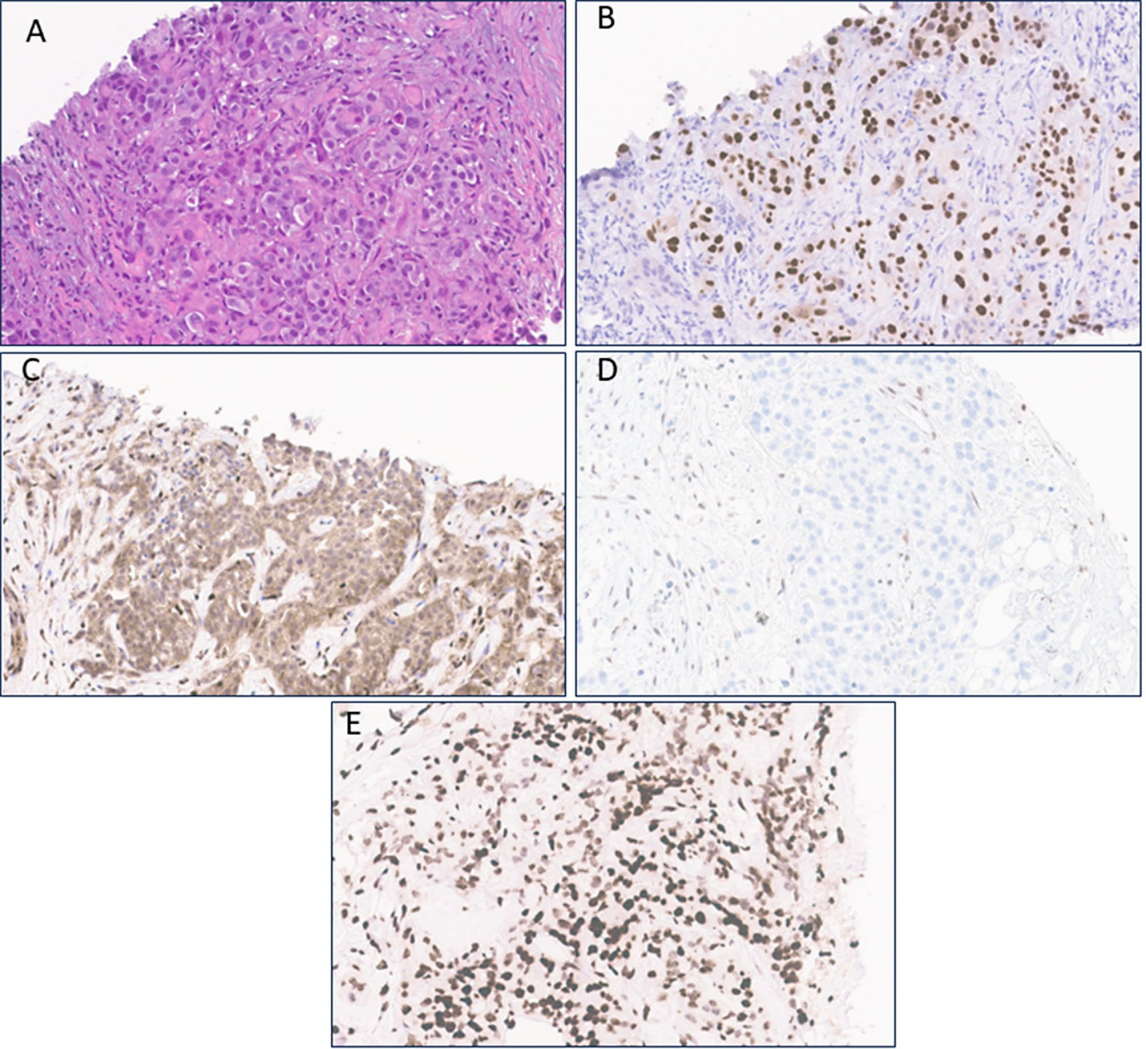
Histomorphologic evaluation of the CT-guided liver biopsy of the metastatic renal medullary carcinoma. **A**, H&E stained section showing atypical epithelioid cells with abundant eosinophilic cytoplasm arranged in glands and embedded in a desmoplastic stroma. **B**, PAX8 shows strong, diffuse nuclear positivity within the neoplastic cells. **C**, INI1 shows high background staining, but demonstrates loss of nuclear expression, similar to the external control, within the tumor cells. **D**, A recent kidney biopsy performed confirms the absence of nuclear INI1 expression in the primary tumor cells with appropriate internal positive control **E**, H3K27me3 stain highlighting intact nuclear expression in both neoplastic cells and background stromal cells

A 21-year-old man with no past medical history, other than regular participation in high-intensity exercise activities, presented with abdominal pain and hematuria. Imaging revealed a 7.4 cm heterogeneous mass centered within the midpole of the right kidney along with hilar and retroperitoneal lymphadenopathy as well as lung and liver lesions, concerning for malignancy. Biopsy of the liver lesion revealed RMC with immunohistochemical stains showing strong expression of PAX8 (Fig. 1A-B). INI-1 was noted to be lost, though there was high background staining (Fig. 1C). INI-1 staining was performed on a later kidney biopsy which showed clearcut evidence of loss of protein expression (Fig. 1D). He was subsequently diagnosed with sickle cell trait by hemoglobin electrophoresis.

The patient was treated with a variety of chemotherapeutic agents including first-line carboplatin plus paclitaxel [26] with disease control for four months, followed by second-line gemcitabine plus doxorubicin [40] with rapid disease progression within two months, then third-line epidermal growth factor receptor (EGFR) inhibition with panitumumab which was combined with nab-paclitaxel [23, 39] resulting in disease response. His disease eventually progressed after 11 months on this third-line regimen, so nab-paclitaxel was accordingly discontinued while EZH2 inhibition with tazemetostat was added to EGFR inhibition with panitumumab. Four months after initiation of tazemetostat, there was slight disease progression for which panitumumab was replaced with proteasome inhibition using bortezomib [14, 27] while tazemetostat was continued.

**Fig. 2:**
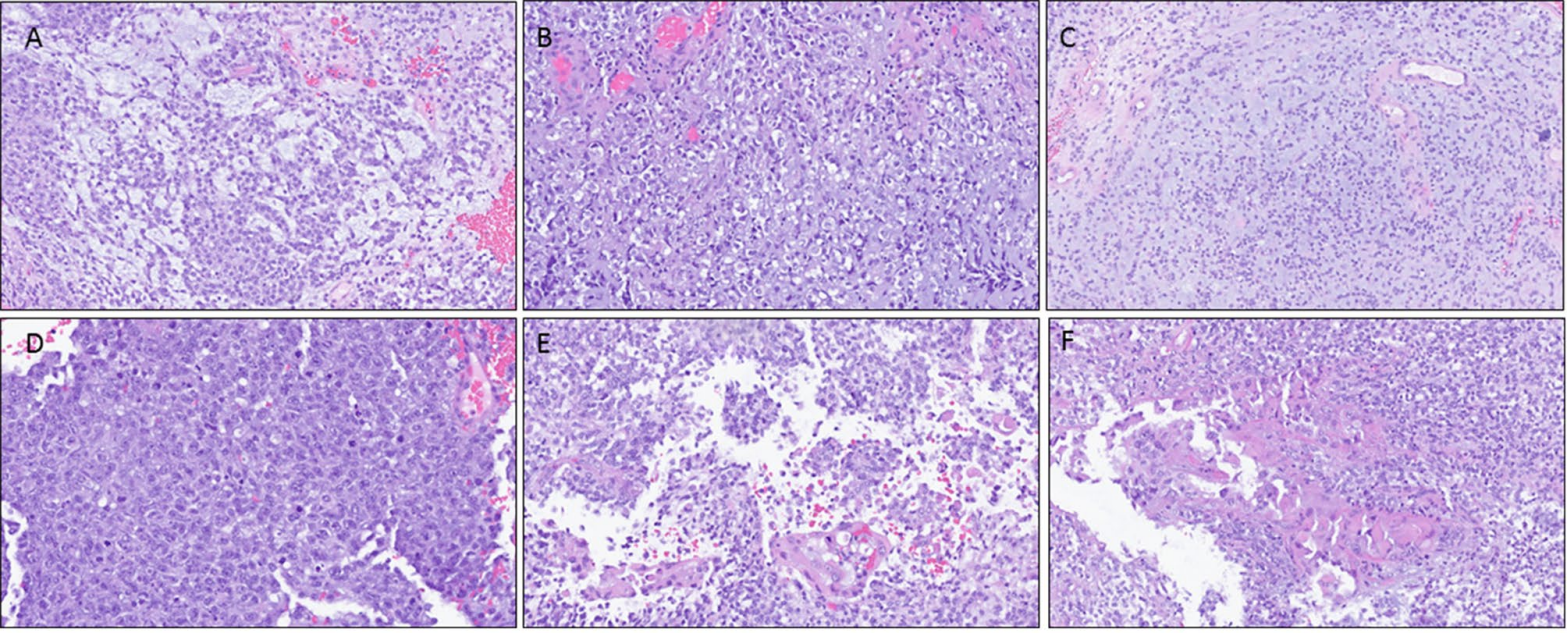
Morphologic appearance of the cerebellar lesion following H&E staining. The undifferentiated tumor shows variable histologic features including **A**, adenoid structures, **B**, large atypical cells with prominent nucleoli and perinuclear clearing, **C**, loosely arranged cells in a myxoid matrix, **D**, densely cellular areas with high mitotic activity, **E**, focal clusters of epithelioid cells with abundant eosinophilic cytoplasm, and **F**, rare cells with abundant eosinophilic cytoplasm imparting a vaguely rhabdoid appearance

**Fig. 3:**
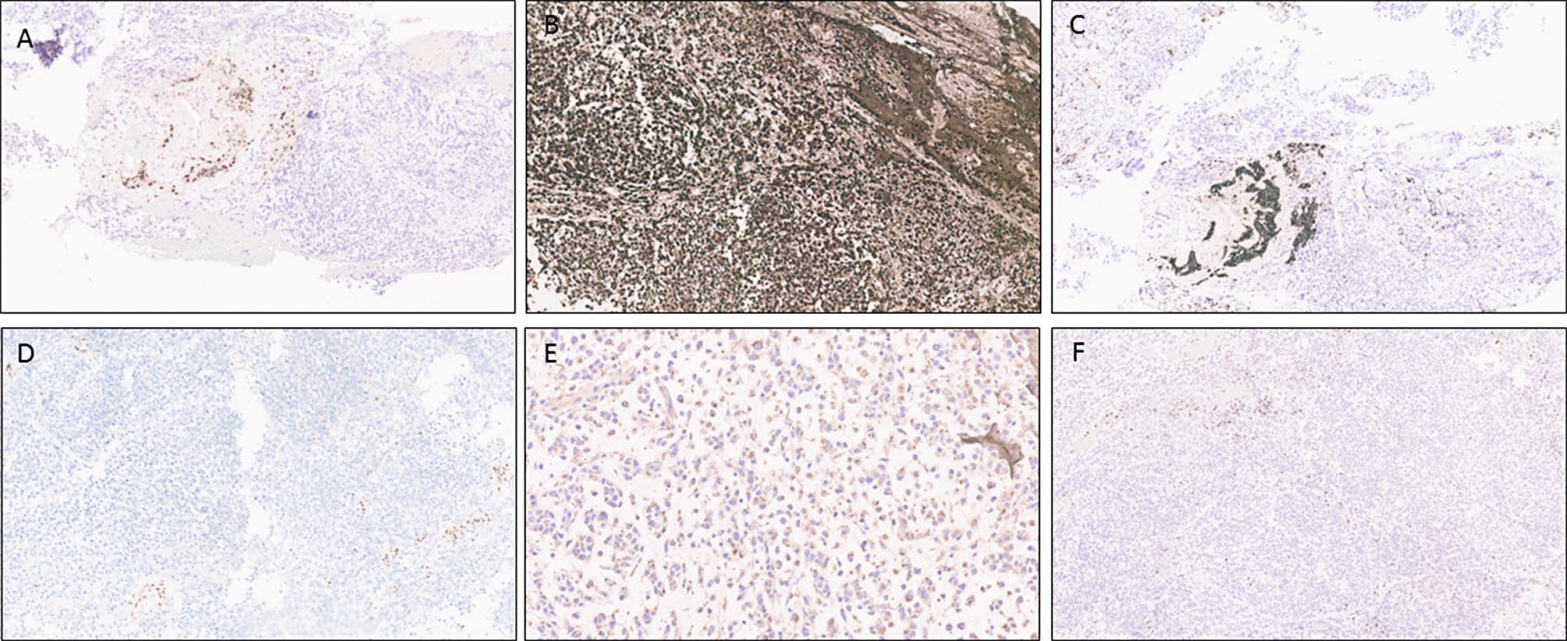
Immunohistochemical studies performed on the cerebellar lesion. **A**, PAX8 is largely negative within the undifferentiated tumor cells but shows nuclear positivity in the regions with epithelioid morphology. **B**, GFAP shows diffuse positivity within the tumor. **C**, Cam5.2 highlights the epithelioid structures. **D**, INI1 expression is lost within the tumor cells with internal positive control showing retained expression in background non-neoplastic tissue. **E**, H3K27M stain is negative. **F**, H3K27me3 stain shows loss of nuclear expression within the tumor cells with internal positive control demonstrating intact expression within the non-neoplastic cells

Approximately one month after the addition of bortezomib to tazemetostat, the patient experienced nausea and vomiting with bilateral lower extremity weakness and headache. Brain imaging revealed a dominant hemorrhagic mass measuring 4.7 × 3.9 × 3.5 cm within the left cerebellum. Additional enhancing lesions were identified within the left temporal lobe and in both parietal and both occipital lobes. Given the multifocality of the lesions and the patient’s history, metastatic disease was suspected. The cerebellar lesion was neurosurgically resected. H&E stained sections showed cerebellar tissue with a well-demarcated border between non-neoplastic cerebellum and tumor. In some areas, the tumor cells were embedded in a myxoid matrix with some foci showing adenoid-type features (Fig. 2A), some demonstrating large, atypical cells with prominent nucleoli and slight perinuclear clearing (Fig. 2B), and others more loosely arranged (Fig. 2C). Scattered regions illustrated more cellular tumor with conspicuous mitotic activity (Fig. 2D). Although the majority of the tumor demonstrated an undifferentiated appearance, focal regions of clusters of more epithelioid-appearing cells with eosinophilic cytoplasm with cell dropout were noted (Fig. 2E) and some areas with a vaguely rhabdoid appearance were also seen (Fig. 2F). Immunohistochemical stains showed the tumor cells were overwhelmingly negative for PAX8; however, the scant clusters of epithelioid cells appeared strongly PAX8 positive (Fig. 3A). GFAP was diffusely positive in the tumor cells and Cam5.2 highlighted scattered cells, including the epithelioid foci (Fig. 3B, C). INI1 expression was lost within the tumor cells with appropriate internal positive control (Fig. 3D). A stain for a mutant histone protein, H3K27M, was negative in the tumor cells (Fig. 3E); however, expression of H3K27me3 was lost in the vast majority of the tumor with satisfactory internal positive control (Fig. 3F).

**Fig. 4:**
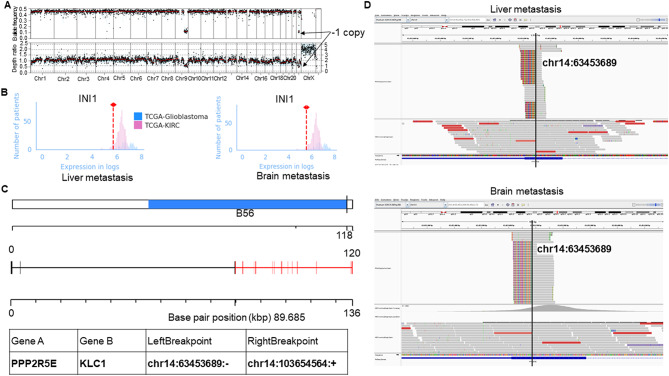
Molecular sequencing of the RMC liver and brain metastases. **A**, Detailed copy number alteration overview of the brain metastasis sample showed partial loss of one copy of chromosome 22 including the *SMARCB1* gene encoding INI1. **B**, RNA expression of INI1 in each of the RMC liver and brain metastasis samples (denoted by the dashed red line in each panel) compared with the distribution of INI1 RNA expression in glioblastoma and clear cell renal cell carcinoma (KIRC) samples from the Cancer Genome Atlas (TCGA). **C**, *PPP2R5E::KLC1* fusion identified in both liver and brain metastasis. Annotation was performed using the AGFusion package. **D**, Integrative genome viewer (IGV) showing that the *PPP2R5E::KLC1* fusion fully corresponds with nucleotide precision in both the liver and brain metastasis samples

Given the patient’s history of metastatic RMC and the radiologic finding of multifocal disease, a metastasis was favored. However, the morphologic appearance of this cerebellar lesion was disparate from that seen in the prior liver metastasis. Moreover, the immunohistochemical profile of the cerebellar lesion, particularly its diffuse GFAP positivity in the absence of significant PAX8 staining brought up the possibility of a primary INI1-deficient CNS neoplasm. To complicate matters further, the loss of H3K27me3 taken together with the midline location raised suspicion for a histone-altered glioma such as a diffuse midline glioma, although such neoplasms do not typically present with INI1 loss in conjunction with histone alterations. To solidify a diagnosis, additional molecular analysis was performed on both the prior liver metastasis and the cerebellar lesion. Whole exome sequencing (WES) and bulk RNA-sequencing (RNA-seq) were performed on formalin-fixed paraffin-embedded (FFPE) tissues as previously described [30]. Both tumors showed downregulation of INI1 (Fig. 4A-B), as expected given the INI1 loss observed by immunohistochemistry in both tumors. Additionally, both tumors demonstrated the presence of a unique fusion, *PPP2R5E::KLC1* (Fig. 4C-D). This fusion was supported by 123 junction reads and 1 spanning read with the exact same breakpoints in both liver and brain samples, supporting that this fusion is not merely an artifact of fixation. The left breakpoint for *PPP2R5E* was found in chr14:63453689 per the Homo sapiens genome assembly GRCh38 (hg38), which is within the coding sequence (CDS) of exon 2 (per NM_001282181.3), whereas the right breakpoint for *KLC1* was in chr14:103654564 which is the 5′ untranslated region (5’UTR) (Fig. 4C). Consequently, this fusion is unlikely to produce a chimeric protein. Furthermore, this fusion is not typically observed in primary glial neoplasms. Importantly, whole exome sequencing and RNA sequencing were performed on the patient’s blood (normal control tissue) and this fusion was not detected, supporting that this alteration is unique to the tumor and not derived from the germline. Therefore, the detection of this identical, likely passenger, molecular alteration in both liver and brain tumor tissues strongly suggested a common origin from the same primary lesion (Fig. 4D). Furthermore, though not meeting quality control threshold, RNA-seq data revealed a *SMARCB1::SREBF2-ASI* fusion in both the liver and brain specimens with identical breakpoints (left breakpoint: chr22:23803408:+, right breakpoint: chr22:41833358:-) (Supplemental Fig. 1). The presence of such fusions is the most common inactivating event for the second *SMARCB1* allele in RMC [28]. A separate passenger fusion, *VPS13B::DEFB1*, also with the same breakpoints in both samples (left breakpoint: chr8:99193057, right breakpoint: chr8:6870826), was additionally identified in the tumor tissues and not the germline sample.

**Fig. 5:**
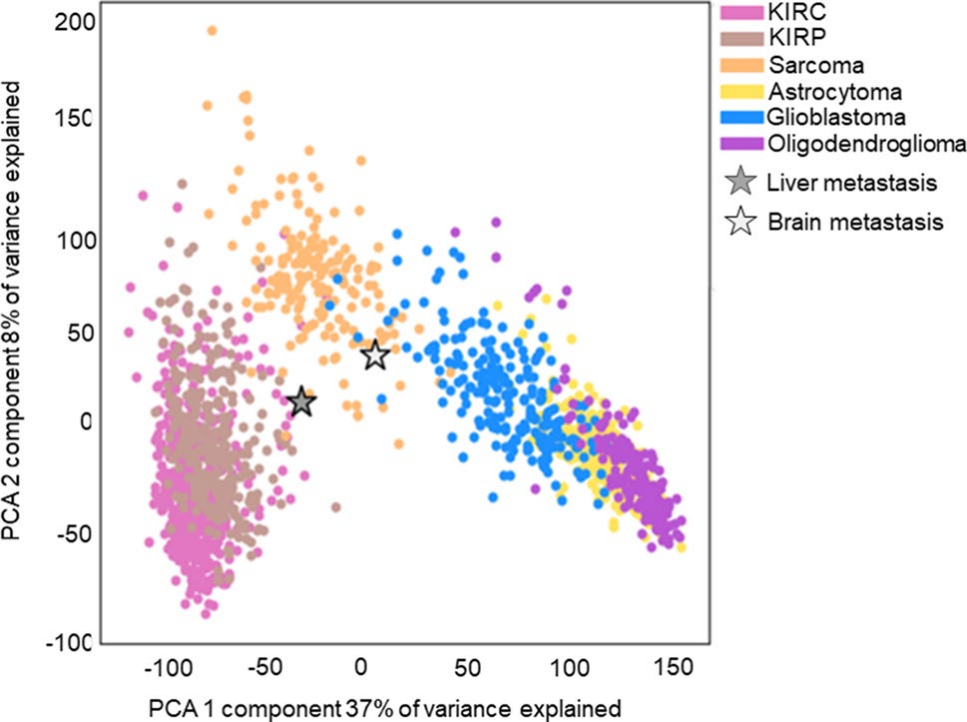
Principal component analysis (PCA) plots of RNA-seq expression in the liver and brain metastasis samples compared with gene expression from the Cancer Genome Atlas (TCGA) of clear cell renal cell carcinoma (KIRC), papillary renal cell carcinoma (KIRP), sarcoma, astrocytoma, glioblastoma and oligodendroglioma samples

DNA methylation profiling was performed on the cerebellar lesion as previously described [41]. DNA methylation-based tumor classification of CNS tumors, using versions 11b6 and 12b6 of the Heidelberg classifier [2], as well as the NCI/Bethesda classifier [41] indicated a consensus match to AT/RT, MYC-activated. The classifiers did not support a diagnosis of diffuse midline glioma. Notably, tumors with INI1 alterations can cluster with the AT/RT group even if they originate outside the CNS [2, 19, 41]. Therefore, while diffuse midline glioma was excluded, it was not possible to determine from the DNA methylation profiling whether the brain tumor represented a metastatic lesion or a new primary. Additional dimensionality reduction with principal component analysis (PCA) was performed using the scikit-learn package version 1.1.1 [30] in RNA-seq from the liver and brain tumors as well as kidney cancer, sarcoma, and CNS tumor samples from the Cancer Genome Atlas (TCGA) with batch correction using Procrustes [21]. This analysis placed the liver tumor closest to the kidney cancer samples whereas the brain tumor was closest to glioblastoma samples (Fig. 5).

Since the patient had received tazemetostat, an EZH2 inhibitor, prior to the discovery of his cerebellar lesion, it was hypothesized that this drug caused the loss of H3K27me3 expression. Immunohistochemical staining for H3K27me3 was retroactively performed on the liver metastasis, which was biopsied before initiation of tazemetostat therapy. This metastatic lesion, which harbored morphologic features classic for RMC, showed retained expression of H3K27me3 (Fig. 1E). Taking all the findings together to include the patient’s clinical history, radiologic findings, comparison of histomorphologic features of his prior and current tumors, and the extensive molecular data, we concluded that this cerebellar lesion was consistent with metastatic disease from the patient’s known RMC with glial mimicry/transdifferentiation. Recent work has identified that upregulation of *PRICKLE1*, a regulator of neuronal development and differentiation, can confer resistance of *SMARCB1*-deficient tumors to tazemetostat [15]. We indeed found that *PRICKLE1* expression was upregulated in the brain metastasis that occurred while the patient was on tazemetostat compared with the liver metastasis collected prior to tazemetostat exposure (Supplementary Table S1).

After concluding that the brain tumor was due to metastatic disease progression and not a new primary CNS malignancy, the patient underwent radiation therapy to the cerebellar cavity and was started on subsequent systemic therapy for metastatic RMC using sacituzumab monotherapy with granulocyte colony stimulating factor (G-CSF) support resulting in improvement of pain control as well as increased general activity level.

### Co-detection by indexing (CODEX) multiplex immunofluorescence

Given the unusual nature of this case, we performed comprehensive immunofluorescence using CODEX as previously described [24] to gain a better understanding of the biology of this rare brain metastasis and to assess its evolution from the prior liver metastasis (Supplemental Figs. 2–5 and Tables 1 and 2). We acknowledge that these studies, while interesting and useful for research purposes, are not clinically validated for diagnosis and are for exploratory characterization only. These assays revealed the tumor cells in the cerebellar lesion showed expression of PAX8 along with focal expression of CA9 (23.7%) and CD10 (4.4%) (Supplemental Fig. 4). Notably, immunohistochemistry for PAX8 was mostly negative (Fig. 3A) suggesting that the signal amplification from the fluorescence-based detection in CODEX was able to detect lower levels of PAX8 expression in the cerebellar metastasis that were not visible using conventional immunohistochemical techniques. More broadly, the tumor cells in the cerebellar metastasis showed focal expression for PanCK (3.5%) and CD56 (13.9%), with a small fraction expressing synaptophysin (1.8%) (Supplemental Fig. 4 and Table 1). In contrast, while similar studies performed on the liver metastasis also showed that the tumor cells expressed PAX8, the percentage of cells expressing other renal markers was markedly higher than in the brain lesion with CA9 expression measured at 77.0% and CD10 expression at 29.5% (Supplemental Fig. 2 and Table 1). Likewise, PanCK showed significantly greater expression in the liver metastasis (84.5%). Synaptophysin expression was not detected in the liver metastasis although CD56 showed weak expression in 46.9% of the cells (Supplemental Fig. 2 and Table 1). The predominant tumor cell type in the brain metastasis, accounting for 65.4% of the tumor cells, was PAX8^+^CA9^−^CD10^−^PanCK^−^CD56^−^Ki-67^+^, and these cells were completely absent in the liver metastasis (Table 2).

**Table 1:** Co-detection by indexing (CODEX) multiplex immunofluorescence of the liver and cerebellar tumors

	Liver tumor	Brain tumor
Tumor cell density	2476.6 per mm^2^	2700.1 per mm^2^
Blood vessel density	124.9 per mm^2^	118.4 per mm^2^
Expression on tumor cells: Ki-67 Synaptophysin CD56 CA9 Pan-CK CD10 PD-L1	38.3% 0% 46.9% 77% 84.5% 29.4% 20%	51.8% 1.9% 13.9% 23.7% 3.5% 4.4% 0.6%
M2 macrophages	68.4%	58.5%
M1/M2 macrophage ratio	0.46	0.7
CD4^+^FOXP3^+^/CD4^+^ ratio	0.12	0.03
CD4^+^FOXP3^+^ cell density	37.4 per mm^2^	9.5 per mm^2^
CD8^+^/CD3^+^ proportion	61.1%	25.8%
CD8^+^ cell density	495.5 per mm^2^	93.7 per mm^2^
PD-L1 on immune cells	7.3%	11.4%
PD-L1^+^ M1 macrophages	13.3%	5%
PD-L1^+^ M2 macrophages	8.4%	7.5%
Proliferating CD8^+^PD-L1^−^ cells	19.4%	30.4%
Proliferating CD8^+^PD-L1^+^ cells	20%	76%
CD4^+^PD-L1^+^ cells	3%	3.3%

**Table 2:** Tumor and immune cell populations by Co-detection by indexing (CODEX) multiplex immunofluorescence of the liver and cerebellar tumors

	Liver tumor	Brain tumor
Tumor cell populations PAX8^+^CD10^+^ PAX8^+^CA9^+^CD56^+^Ki-67^−^ PAX8^+^CA9^+^CD56^+^Ki-67^+^ PAX8^+^CD10^+^CD56^+^ PAX8^+^CA9^+^Ki-67^−^ PAX8^+^CA9^+^Ki-67^+^ PAX8^+^CD10^+^Ki-67^+^ PAX8^+^CD10^+^Ki-67^−^ PAX8^+^CD56^+^Ki-67^+^ PAX8^+^CD10^+^CD56^+^Ki-67^+^ PAX8^+^CA9^−^CD10^−^PanCK^−^CD56^−^Ki-67^+^ PAX8^+^CA9^−^CD10^−^PanCK^−^CD56^+^Ki-67^−^ PanCK^+^CA9^+^ PanCK^+^CA9^+^Ki-67^+^ PanCK^+^CA9^+^CD56^+^ PanCK^+^CA9^+^CD56^+^Ki-67^+^	10.7% 21.7% 23.4% 1.9% 7.2% 6.3% 1.5% 0% 0% 1.0% 0% 0% 17.3% 5.8% 2.8% 0.5%	0% 0% 2% 0% 0% 8.8% 1.5% 16.4% 5.2% 0% 65.4% 0.8% 0% 0% 0% 0%
Immune cell populations CD68^+^ CD163^−^CD206^−^ (M1 macrophages) CD68^−^/^+^CD163/CD206^+^ (M2 macrophages) CD4^+^ CD8^+^ CD4^+^FOXP3^+^ CD11b^+^PD-L1^−^ CD45^+^	16.5% 35.8% 16.3% 29.2% 2.2% 0% 0%	22.6% 31.8% 26.7% 9.3% 0.9% 0.4% 8.2%

As previously described [31], CD68^+^CD163^−^CD206^−^ macrophages were classified as M1 proinflammatory, whereas CD68^−/+^CD163/CD206^+^ macrophages were classified as M2 anti-inflammatory. Assessment of the tumor microenvironment of the brain metastasis revealed a mixture of inflammatory cells with a concentration of both M1 and M2 macrophages clustering both at the border of the tumor as well as within the tumor proper (Supplemental Fig. 5). To a lesser extent, T lymphocytes were seen in a similar distribution. Of interest, only a small percentage of tumor cells expressed PD-L1 (< 1%) whereas 11.4% of the immune cells showed expression (Supplemental Fig. 4 and Table 1). In the liver metastasis, M2 macrophages were the predominant phenotype and were mostly found in regions of desmoplasia and high microvessel density (Supplemental Fig. 3 and Table 2). M1 macrophages were mostly seen in the dense regions of the tumor. PD-L1 expression was higher in the liver metastasis tumor cells vis-à-vis the cerebellar tumor cells and was estimated at 20% (Supplemental Fig. 3 and Table 1). Overall, the liver metastasis exhibited lower proliferation rate by Ki-67 (38.3% versus 51.8%), higher density of both CD8 + T lymphocytes (495.5 cells per mm^3^ versus 93.7 cells per mm^3^) and CD4 + FOXP3 + T lymphocytes (37.4 cells per mm^3^ versus 9.5 cells per mm^3^), as well as higher tumor cell diversity when compared with the brain metastasis (Supplemental Fig. 3A, 4 A and Table 2).

## Discussion

We present an exceedingly rare scenario of brain metastasis from RMC. Only scattered reports of this phenomenon can be found in the literature [3, 23, 32, 37]. It is unclear from those prior studies whether the metastatic tumor retained its renal medullary phenotype as most of those brain lesions were not biopsied or surgically resected for histopathologic review. To date, glial transdifferentiation in metastatic RMC has not been reported making our case the first documented occurrence. In that vein, RMC has been known to exhibit extraordinary levels of cell plasticity, including hyperprogression due to myeloid mimicry following immune checkpoint inhibitor therapy [34]. One proposed hypothesis for this ability to mimic other cell types is that the loss of the epigenetic chromatin remodeler INI1 releases the restriction to specific cell states [28]. H3K27me3 serves as another critical mechanism for maintaining transcriptional regulatory programs [5]. Consequently, the loss of H3K27me3 through EZH2 inhibition, as observed in our case, may further promote the co-option of different cellular states.

We hypothesize that the high plasticity of RMC in conjunction with EZH2 inhibition by tazemetostat allowed tumor cells to metastasize to the CNS by acquiring a glial phenotype imparting a survival advantage in the unique milieu of the cerebellum. Interestingly, a similar situation has been reported in breast cancer whereby tumor cells can utilize the tumor microenvironment of the bone marrow to promote survival and spread to the more hostile leptomeningeal environment via borrowing neuronal signaling pathways [38]. In addition, glioblastoma, a primary brain tumor, can undergo myeloid mimicry following epigenetic modulation in order to evade destruction via immunotherapy [9]. More work needs to be done to elucidate the mechanistic steps of cell mimicry, both with regard to tumors mimicking non-neoplastic cell residents as well as, in our case, a tumor mimicking a distinctly separate tumor type.

Though rare, other tumors, most strikingly melanoma, have been known to transdifferentiate and the transdifferentiation is often only revealed following thorough analysis at the molecular level [17, 20]. We performed comprehensive studies to confirm that our case was a bona fide example of transdifferentiation. First, our extensive molecular profiling revealed shared genetic alterations in both the liver and brain metastases, including the likely non-functional *PPP2R5E::KLC1* and *VPS13B::DEFB1* fusions, and the presence of the same *SMARCB1::SREBF2-AS1* fusion. Notably such *SMARCB1* translocation events are highly prevalent in RMC [28]. Though whole exome sequencing of sufficient quality could not be obtained from the liver sample despite multiple attempts, we were able to compare the somatic mutations identified in the brain sample with the limited data in the liver sample, revealing traces of them in the liver metastasis. Despite these limitations, these findings strengthen our assertion of clonal relationships between the samples. Next, our comprehensive immunohistochemical and immunofluorescent studies revealed that, despite these shared genetic signatures, the brain and liver metastases showed disparate expression patterns of inflammatory, glial, renal, and hypoxic biomarkers, supporting inherent differences between the two tumors. For the brain metastasis, DNA methylation profiling showed a consensus match to the class of AT/RT, MYC-activated. Unfortunately, there was insufficient tissue available from the biopsy prior to initiation of tazemetostat therapy to perform comparative DNA methylation profiling studies. We acknowledge this is a limitation to this study as direct comparison of the DNA methylation profiles of the tumor pre-treatment and post-treatment would have been helpful. Of note, DNA methylation profiling often cannot differentiate specific *SMARCB1*-altered tumors from one another as such tumors tend to cluster within the AT/RT group. While this is a separate limitation of this study pertaining specifically to methylation profiling, the fact that this particular tumor clustered to the MYC-activated subtype of AT/RT is significant as RMC is particularly enriched for MYC [28]. Thus, it is not surprising that if our tumor were to match to a particular subset of AT/RT, the MYC-activated subtype should be the class where it best belongs.

We must also mention one additional piece of information regarding the loss of H3K27me3 expression in this patient’s brain lesion. The loss of H3K27me3 expression is commonly seen in primary CNS neoplasms such as posterior fossa ependymoma and in diffuse midline glioma, H3K27-altered, but has also been reported in AT/RT [12, 36]. Though it is possible for AT/RT to harbor an immunohistochemical profile such as this tumor, we strongly favor this tumor to be a metastasis of RMC with loss of H3K27me3 related to tazemetostat therapy given the multifocal nature of the brain lesions (AT/RT are rarely if ever multifocal), the shared molecular alterations between the brain lesion and liver metastasis, and the fact that AT/RT and RMC have never been reported to co-occur.

Our findings raise concern that EZH2 inhibition by drugs such as tazemetostat, either alone or in combination with other agents such as the EGFR inhibitor panitumumab or the proteasome inhibitor bortezomib, as used in our case, may increase the risk of CNS metastasis through glial mimicry in INI1-deficient tumors such as RMC and epithelioid sarcoma. Given the loss of H3K27me3 in the brain metastasis and the intact nature of this protein in the liver metastasis (obtained prior to initiation of tazemetostat therapy), we speculate that this drug altered the epigenetic composition of this tumor. While we cannot say with certainty that these epigenetic changes permitted the metastasis and subsequent transdifferentiation of this tumor, we hypothesize that, at the very least, it significantly contributed. Therefore, the routine use of this drug in patients with RMC may need careful assessment of risks and benefits. Further analysis of other patients with RMC who have undergone treatment with this drug is warranted. Given the regulatory approval of tazemetostat as a salvage therapy in patients with epithelioid sarcoma, close monitoring will also be required in patients with these INI1-deficient tumors to ensure that they are unable to undergo glial mimicry.

In summary, we report an extremely rare finding of glial mimicry/transdifferentiation of metastatic RMC in a patient following treatment with the EZH2 inhibitor, tazemetostat. We bring this case to the literature to contribute our experience and provoke further investigation of such unexpected and profound cellular plasticity, particularly in response to therapeutic intervention.

## Electronic supplementary material

Below is the link to the electronic supplementary material.

Supplementary Material 1: Supplemental Fig. 1: Copy number alteration overview of the brain metastasis sample showing the locations of the partial loss of one *SMARCB1* allele and the coordinates of the fusion breakpoints for the *SMARCB1::SREBF2-AS1* fusion. The top panel shows the total allele copies (black line) and minor allele copies (red line). The middle panel presents the ratio of tumor to normal depth coverage at specific positions. The bottom panel displays the B allele frequency (BAF) for heterozygous variants, representing the frequency of the minor allele. The differing breakpoints of the *SMARCB1* deletion and fusion further suggest that each event occurred in a separate *SMARCB1* allele.

Supplementary Material 2: Supplemental Fig. 2: Co-Detection by Indexing (CODEX) multiplex immunofluorescence of the liver metastasis. **A**, Morphologic appearance following H&E staining of the liver metastasis sample. The sample consisted of a single core biopsy. No fragments of normal liver tissue were present. **B**, Tumor cell subtyping. Tumor cells, identified by the expression of the PAX8 marker, made up 43.8% of all cells, immune cells constituted 26.7%, and stromal cells represented 30.0% of the total cell population. Most tumor cells showed elevated expression of CA9 (77.0%) and pan-CK (84.5%), whereas PD-L1 (20.1%), CD10 (29.5%), ki-67 (38.3%), and CD56 (46.9%) were increase in a smaller subset of tumor cells. Synaptophysin expression was not detected. **C**, Tumor microenvironment cell subtyping. A high proportion (11.8%) of CD4 + FOXP3 + T cells was found whereas only 3.0% of all CD4 + cells expressed PD-L1. CD8 + T cells were more abundant than CD4 + T cells (CD8+/CD3 + proportion of 61.1%). Of the CD8 + T cells, 28.8% expressed Granzyme B, while only 3.4% expressed PD-L1. Pronounced stromal reaction was noted harboring a high microvessel density (124.9 per mm²), a large number of fibronectin (FN1) fibers and SMA + fibers, particularly in areas with desmoplastic reaction.

Supplementary Material 3: Supplemental Fig. 3: Spatial distribution of tumor cells **(A)** and immune cells **(B)** by Co-Detection by Indexing (CODEX) multiplex immunofluorescence of the liver metastasis. M1 macrophages (CD68 + CD163-CD206-) were predominantly localized in areas with higher tumor cell density, while M2 macrophages (CD68+/- CD163/CD206+) were found in regions with greater microvessel density and desmoplastic stromal reaction. Nearly all CD4 + T-lymphocytes were concentrated in the central core area near the tumor, whereas CD8 + cells were distributed more evenly throughout the tissue. Colorbar density description in Supplementary Table S2.

Supplementary Material 4: Supplemental Fig. 4: Co-Detection by Indexing (CODEX) multiplex immunofluorescence of the resected brain tumor. **A**, Morphologic appearance following H&E staining of the resected cerebellar tumor. **B**, Tumor cell subtyping. Tumor cells, identified by the expression of the PAX8 marker, made up 84% of the total cell population. Ki-67 was expressed in 51.8% of tumor cells, while a smaller proportion (23.7%) of tumor cells expressed CA9, and there was only weak expression of pan-CK (3.5%), PD-L1 (0.6%), CD10 (4.4%), and CD56 (13.9%). Synaptophysin expression was detected in 1.8% of PAX8 + tumor cells. **C**, Tumor microenvironment cell subtyping. A high proportion (11.4%) of immune cells expressed PD-L1. CD4 + FOXP3 + T cells accounted for 3.4% of the total CD4 + cell population. CD8 + T cells were less prevalent than CD4 + T cells (CD8+/CD3 + proportion of 25.8%). Of the CD8 + T cells, 18.1% expressed Granzyme B, while 19.5% expressed PD-L1. The tumor exhibited a high microvessel density (118.4 per mm²).

Supplementary Material 5: Supplemental Fig. 5: Spatial distribution of tumor cells **(A)** and immune cells **(B)** by co-Detection by Indexing (CODEX) multiplex immunofluorescence of the resected brain tumor. The spatial distribution of macrophages shows their concentration both at the borders of the tumor tissue and within it, where they form relatively compact clusters. This pattern is observed for both M1 (CD68 + CD163-CD206-) and M2 (CD68+/- CD163/CD206+) macrophages. CD3 + T lymphocytes were present in smaller numbers but exhibit a similar distribution pattern, suggesting close interactions between these immune cell types. Colorbar density description in Supplementary Table S3.

Supplementary Material 6

## Data Availability

No datasets were generated or analysed during the current study.

## Abbreviations

CNS: Central Nervous System
EGFR: Epidermal Growth Factor Receptor
EZH2: Enhancer of Zeste Homolog 2
G-CSF: Granulocyte Colony Stimulating Factor
H3K27me3: Trimethylated lysine 27 on Histone 3
H3K27M: Mutant Histone 3 lysine 27 to Methionine
INI-1: Integrase Interactor 1
RMC: Renal Medullary Carcinoma
SMARCB1: Switch/Sucrose Nonfermenting Matrix Associated Actin Dependent Regulator of Chromatin, Subfamily B, Member 1
