# Supplementary figures and images for: A novel case of glial transdifferentiation in renal medullary carcinoma brain metastasis
Source: Acta Neuropathol Commun. 2025 Jan 20;13:12. doi: 10.1186/s40478-025-01929-w (PMC11748356; doi:10.1186/s40478-025-01929-w)

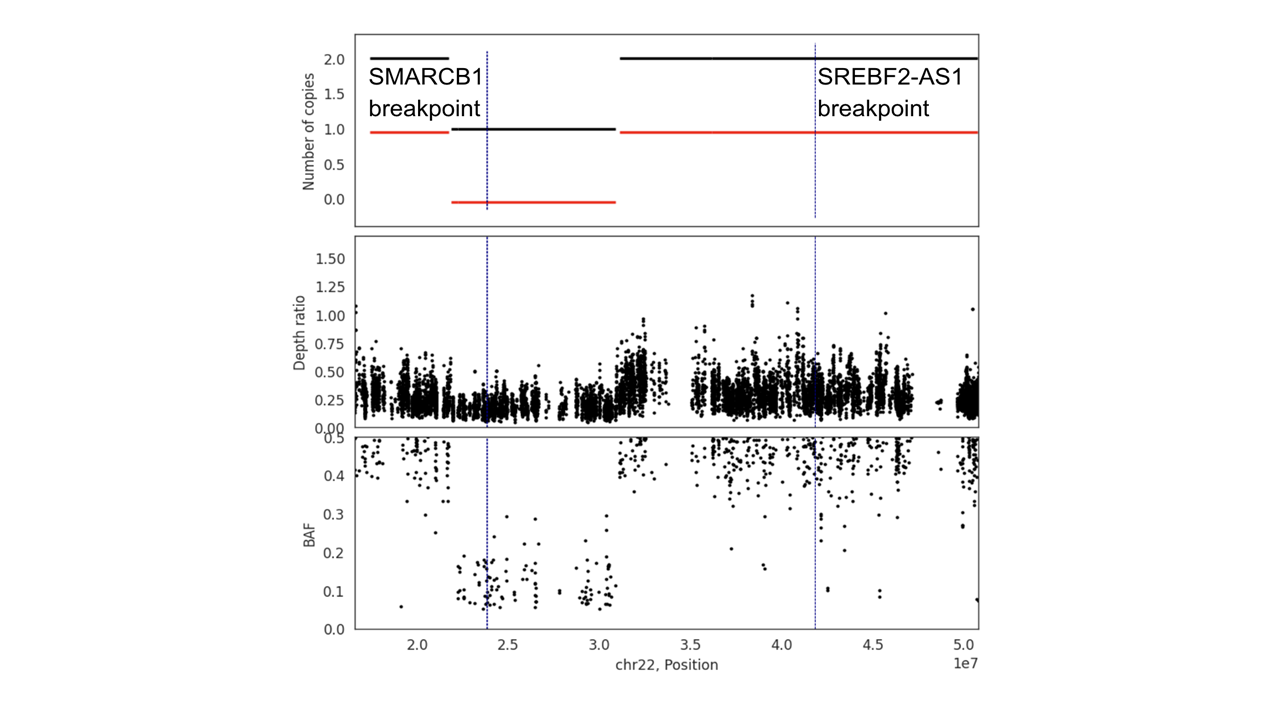

Supplement: Supplementary file 1 — Supplementary Material 1: Supplemental Fig. 1: Copy number alteration overview of the brain metastasis sample showing the locations of the partial loss of one SMARCB1 allele and the coordinates of the fusion breakpoints for the SMARCB1::SREBF2-AS1 fusion. The top panel shows the total allele copies (black line) and minor allele copies (red line). The middle panel presents the ratio of tumor to normal depth coverage at specific positions. The bottom panel displays the B allele frequency (BAF) for heterozygous variants, representing the frequency of the minor allele. The differing breakpoints of the SMARCB1 deletion and fusion further suggest that each event occurred in a separate SMARCB1 allele. [file 40478_2025_1929_MOESM1_ESM.tif]

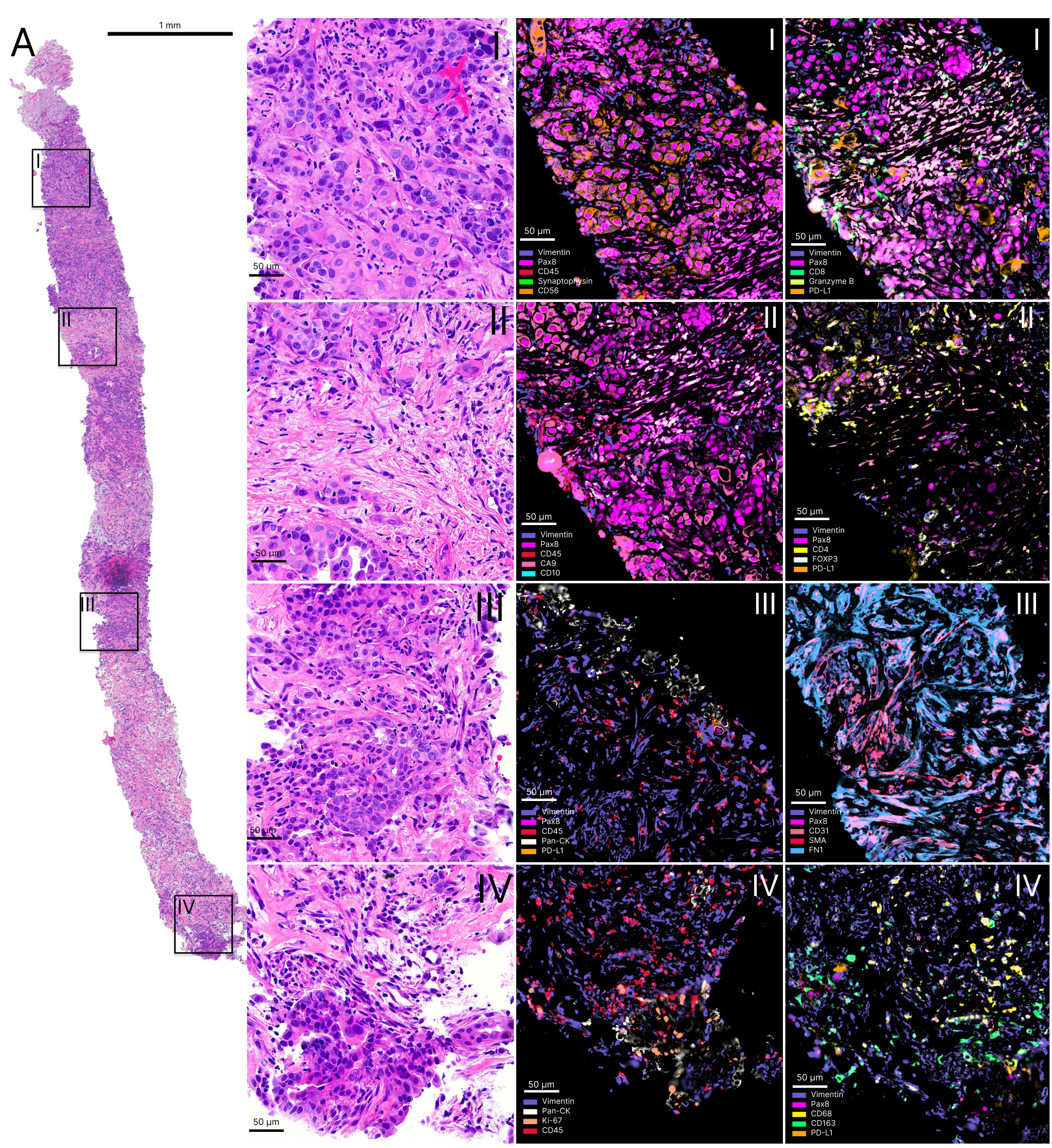

Supplement: Supplementary file 2 — Supplementary Material 2: Supplemental Fig. 2: Co-Detection by Indexing (CODEX) multiplex immunofluorescence of the liver metastasis. A, Morphologic appearance following H&E staining of the liver metastasis sample. The sample consisted of a single core biopsy. No fragments of normal liver tissue were present. B, Tumor cell subtyping. Tumor cells, identified by the expression of the PAX8 marker, made up 43.8% of all cells, immune cells constituted 26.7%, and stromal cells represented 30.0% of the total cell population. Most tumor cells showed elevated expression of CA9 (77.0%) and pan-CK (84.5%), whereas PD-L1 (20.1%), CD10 (29.5%), ki-67 (38.3%), and CD56 (46.9%) were increase in a smaller subset of tumor cells. Synaptophysin expression was not detected. C, Tumor microenvironment cell subtyping. A high proportion (11.8%) of CD4 + FOXP3 + T cells was found whereas only 3.0% of all CD4 + cells expressed PD-L1. CD8 + T cells were more abundant than CD4 + T cells (CD8+/CD3 + proportion of 61.1%). Of the CD8 + T cells, 28.8% expressed Granzyme B, while only 3.4% expressed PD-L1. Pronounced stromal reaction was noted harboring a high microvessel density (124.9 per mm²), a large number of fibronectin (FN1) fibers and SMA + fibers, particularly in areas with desmoplastic reaction. [file 40478_2025_1929_MOESM2_ESM.jpeg]

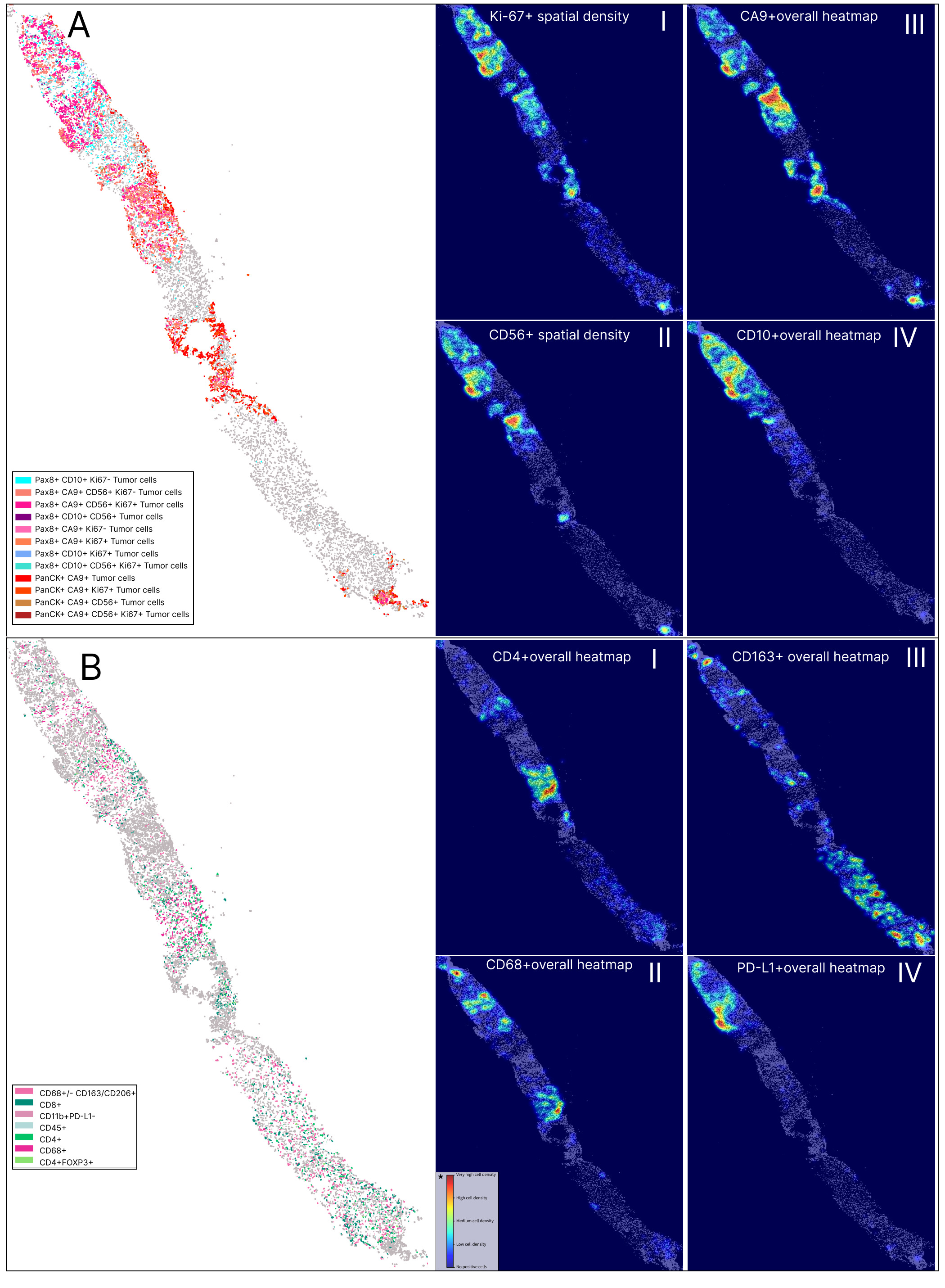

Supplement: Supplementary file 3 — Supplementary Material 3: Supplemental Fig. 3: Spatial distribution of tumor cells (A) and immune cells (B) by Co-Detection by Indexing (CODEX) multiplex immunofluorescence of the liver metastasis. M1 macrophages (CD68 + CD163-CD206-) were predominantly localized in areas with higher tumor cell density, while M2 macrophages (CD68+/- CD163/CD206+) were found in regions with greater microvessel density and desmoplastic stromal reaction. Nearly all CD4 + T-lymphocytes were concentrated in the central core area near the tumor, whereas CD8 + cells were distributed more evenly throughout the tissue. Colorbar density description in Supplementary Table S2. [file 40478_2025_1929_MOESM3_ESM.jpeg]

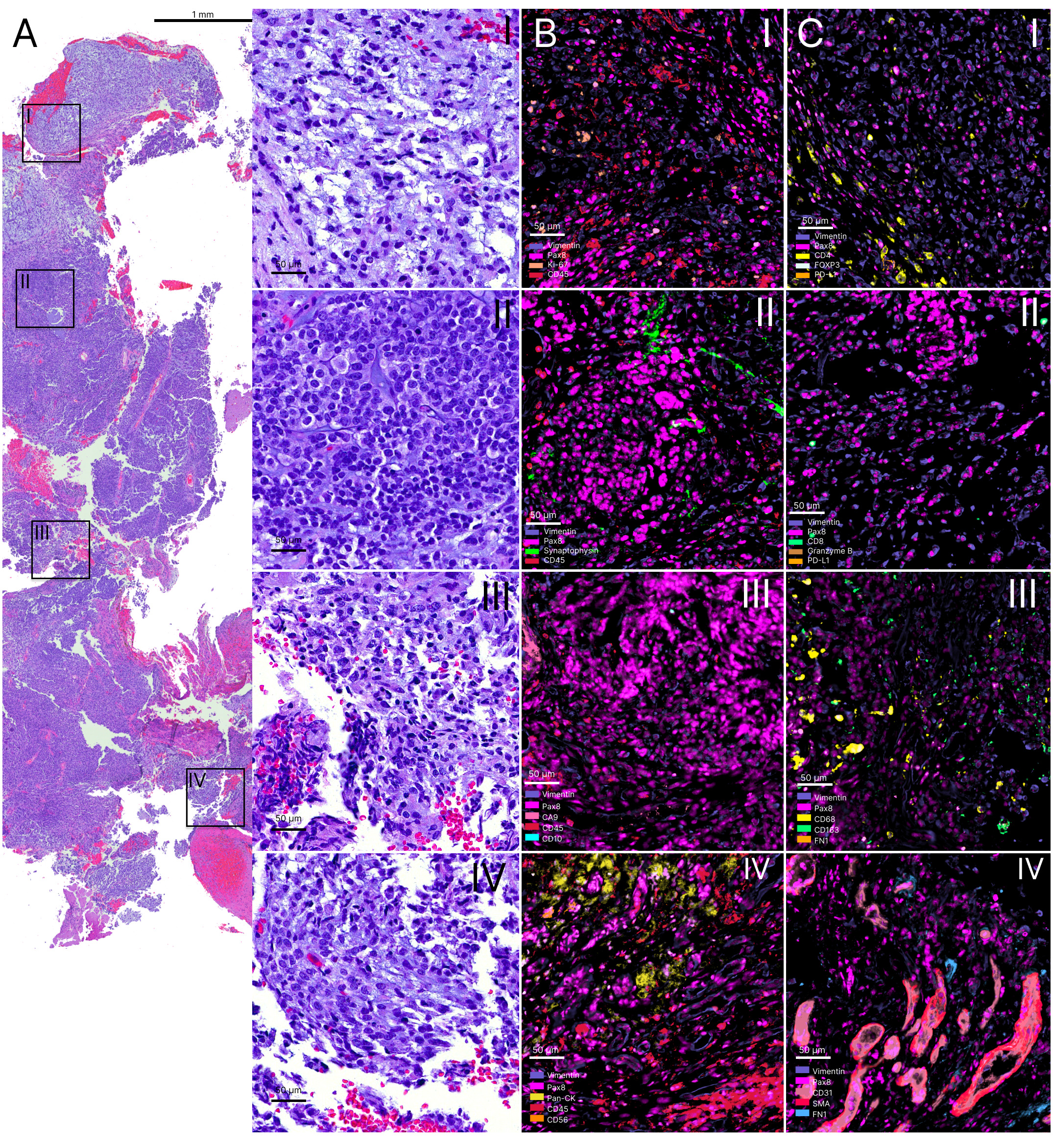

Supplement: Supplementary file 4 — Supplementary Material 4: Supplemental Fig. 4: Co-Detection by Indexing (CODEX) multiplex immunofluorescence of the resected brain tumor. A, Morphologic appearance following H&E staining of the resected cerebellar tumor. B, Tumor cell subtyping. Tumor cells, identified by the expression of the PAX8 marker, made up 84% of the total cell population. Ki-67 was expressed in 51.8% of tumor cells, while a smaller proportion (23.7%) of tumor cells expressed CA9, and there was only weak expression of pan-CK (3.5%), PD-L1 (0.6%), CD10 (4.4%), and CD56 (13.9%). Synaptophysin expression was detected in 1.8% of PAX8 + tumor cells. C, Tumor microenvironment cell subtyping. A high proportion (11.4%) of immune cells expressed PD-L1. CD4 + FOXP3 + T cells accounted for 3.4% of the total CD4 + cell population. CD8 + T cells were less prevalent than CD4 + T cells (CD8+/CD3 + proportion of 25.8%). Of the CD8 + T cells, 18.1% expressed Granzyme B, while 19.5% expressed PD-L1. The tumor exhibited a high microvessel density (118.4 per mm²). [file 40478_2025_1929_MOESM4_ESM.jpeg]

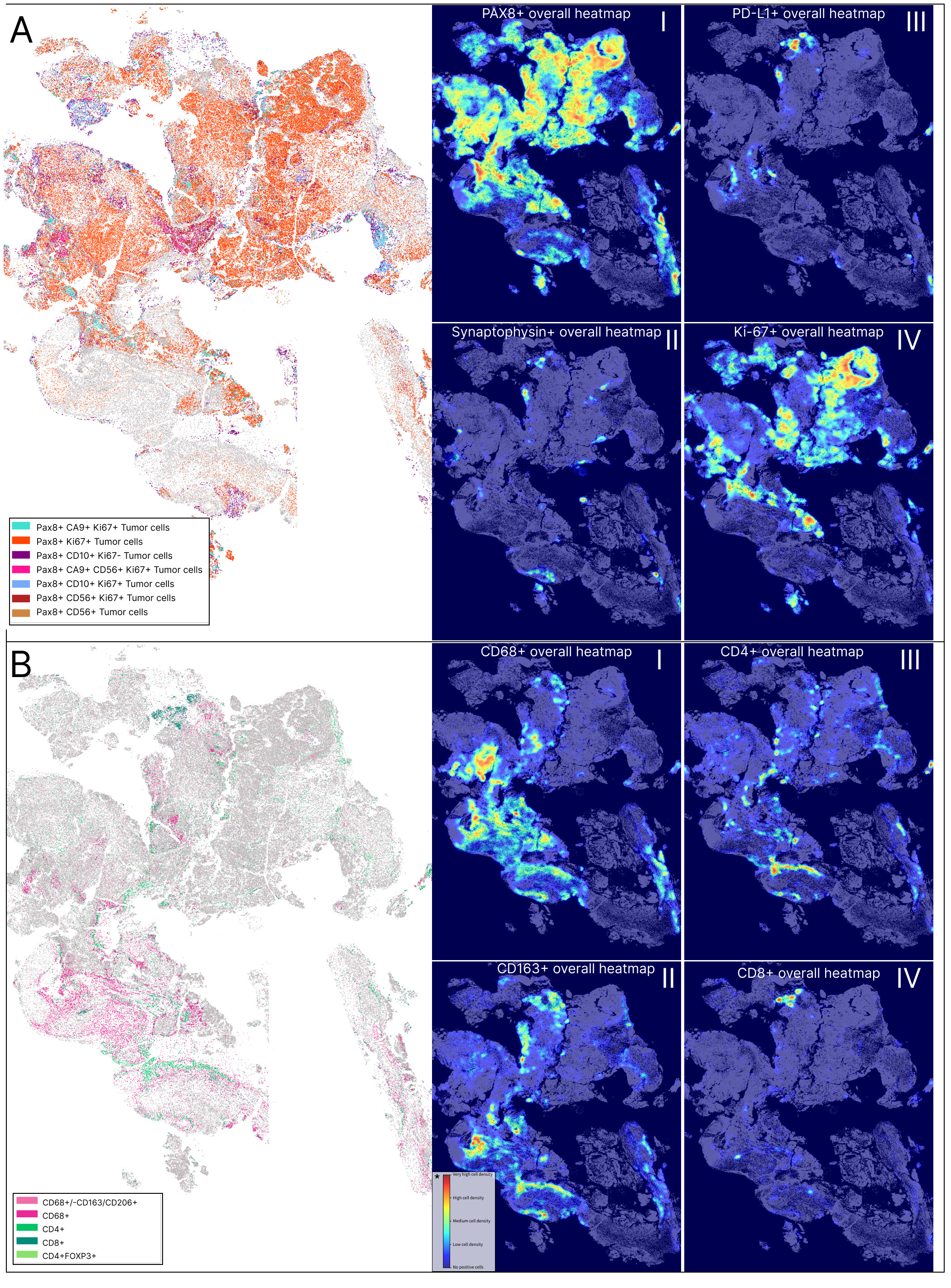

Supplement: Supplementary file 5 — Supplementary Material 5: Supplemental Fig. 5: Spatial distribution of tumor cells (A) and immune cells (B) by co-Detection by Indexing (CODEX) multiplex immunofluorescence of the resected brain tumor. The spatial distribution of macrophages shows their concentration both at the borders of the tumor tissue and within it, where they form relatively compact clusters. This pattern is observed for both M1 (CD68 + CD163-CD206-) and M2 (CD68+/- CD163/CD206+) macrophages. CD3 + T lymphocytes were present in smaller numbers but exhibit a similar distribution pattern, suggesting close interactions between these immune cell types. Colorbar density description in Supplementary Table S3. [file 40478_2025_1929_MOESM5_ESM.jpeg]
