# Supplementary material for: A novel case of glial transdifferentiation in renal medullary carcinoma brain metastasis
Source: Acta Neuropathol Commun. 2025 Jan 20;13:12. doi: 10.1186/s40478-025-01929-w (PMC11748356; doi:10.1186/s40478-025-01929-w)
Supplement: Supplementary file 6 — Supplementary Material 6 [file 40478_2025_1929_MOESM6_ESM.docx]

**SUPPLEMENTARY TABLES**

| **Supplementary Table S1.** Gene expression in the liver and brain tumor metastases quantified as transcripts per million (TPM) and log2-transformed. Gene expression ranking is also shown in comparison with the reference PanSolid cohort sequenced by BostonGene similarly to liver and brain metastasis samples. | | | | |
| --- | --- | --- | --- | --- |
| **Gene** | **Liver metastasis, log2(1+TPM)** | **Brain metastasis, log2(1+TPM)** | **Liver metastasis PanSolid cohort rank** | **Brain metastasis PanSolid cohort rank** |
| *PRICKLE1* | 5.61 | 7.65 | 61 | 96 |

| **Supplementary Table S2.** Liver metastasis density heatmap description. | | | | |
| --- | --- | --- | --- | --- |
| **Biomarker** | **25th percentile** | **50th percentile** | **75th percentile** | **100th percentile** |
| **Ki-67+** | 8 | 16 | 32 | 112 |
| **CA9+** | 16 | 32 | 48 | 144 |
| **CD10+** | 8 | 16 | 32 | 112 |
| **CD56+** | 16 | 32 | 48 | 128 |
| **CD4+** | 8 | 16 | 32 | 96 |
| **CD68+** | 8 | 16 | 32 | 112 |
| **CD163+** | 8 | 16 | 32 | 48 |
| **PD-L1+** | 8 | 16 | 32 | 112 |
| For each spatial heatmap small tiles of ~100x115 micrometers were used within which cell count with positive expression of marker was calculated. Obtained cell count was then rescaled to match “density per mm^2^” unit. Heatmap zones were color coded based on density percentiles (25th percentile - low, 50th percentile - medium, 75th percentile - high, 100th percentile - very high) within all heatmap values. | | | | |

| **Supplementary Table S3.** Brain metastasis density heatmap description. | | | | |
| --- | --- | --- | --- | --- |
| **Biomarker** | **25th percentile** | **50th percentile** | **75th percentile** | **100th percentile** |
| **Ki-67+** | 32 | 80 | 176 | 704 |
| **Pax8+** | 80 | 192 | 336 | 880 |
| **Synaptophysin+** | 8 | 16 | 48 | 480 |
| **PD-L1+** | 8 | 16 | 64 | 576 |
| **CD4+** | 16 | 32 | 48 | 352 |
| **CD8+** | 8 | 16 | 32 | 368 |
| **CD68+** | 16 | 48 | 144 | 688 |
| **CD163+** | 16 | 32 | 64 | 432 |
| For each spatial heatmap small tiles of ~100x115 micrometers were used within which cell count with positive expression of marker was calculated. Obtained cell count was then rescaled to match “density per mm^2^” unit. Heatmap zones were color coded based on density percentiles (25th percentile - low, 50th percentile - medium, 75th percentile - high, 100th percentile - very high) within all heatmap values. | | | | |
